# Phi29 polymerase based random amplification of viral RNA as an alternative to random RT-PCR

**DOI:** 10.1186/1471-2199-9-77

**Published:** 2008-09-04

**Authors:** Nicolas Berthet, Anita K Reinhardt, India Leclercq, Sven van Ooyen, Christophe Batéjat, Philip Dickinson, Rayna Stamboliyska, Iain G Old, Katherine A Kong, Laurent Dacheux, Hervé Bourhy, Giulia C Kennedy, Christian Korfhage, Stewart T Cole, Jean-Claude Manuguerra

**Affiliations:** 1 Genotyping of Pathogens and Public Health Technological Platform, Institut Pasteur, Paris, France; 2 Oncogenic Virus Epidemiology and Pathophysiology Unit, Institut Pasteur, Paris, France; 3 Laboratory for Urgent Response to Biological Threats (CIBU), Institut Pasteur, Paris, France; 4 Université Paris 7 Denis Diderot, Paris, France; 5 Qiagen, Hilden, Germany; 6 Affymetrix, Santa Clara, CA, USA; 7 European Office, Institut Pasteur, Paris, France; 8 Lyssavirus dynamics and host adaptation Unit, Institut Pasteur, Paris, France; 9 Bacterial Molecular Genetics Unit, Institut Pasteur, Paris, France

## Abstract

**Background:**

Phi29 polymerase based amplification methods provides amplified DNA with minimal changes in sequence and relative abundance for many biomedical applications. RNA virus detection using microarrays, however, can present a challenge because phi29 DNA polymerase cannot amplify RNA nor small cDNA fragments (<2000 bases) obtained by reverse transcription of certain viral RNA genomes. Therefore, ligation of cDNA fragments is necessary prior phi29 polymerase based amplification. We adapted the QuantiTect Whole Transcriptome Kit (Qiagen) to our purposes and designated the method as Whole Transcriptome Amplification (WTA).

**Results:**

WTA successfully amplified cDNA from a panel of RNA viruses representing the diversity of ribovirus genome sizes. We amplified a range of genome copy numbers from 15 to 4 × 10^7 ^using WTA, which yielded quantities of amplified DNA as high as 1.2 μg/μl or 10^10 ^target copies. The amplification factor varied between 10^9 ^and 10^6^. We also demonstrated that co-amplification occurred when viral RNA was mixed with bacterial DNA.

**Conclusion:**

This is the first report in the scientific literature showing that a modified WGA (WTA) approach can be successfully applied to viral genomic RNA of all sizes. Amplifying viral RNA by WTA provides considerably better sensitivity and accuracy of detection compared to random RT-PCR.

## Background

Parallelization of nucleic acid sequence detection requires a sufficient quantity (in the microgram range) of DNA for subsequent hybridization-based methods such as those using DNA microarrays or resequencing arrays. For RNA virus detection, target DNA represents the amplified product of reverse transcription (RT). RT and DNA amplification can be achieved either by using primers specific for relevant viruses or by random priming. Although random priming can amplify an unknown target, it often yields lower amounts of DNA than specific primers, which can reduce the overall sensitivity of the process. Multiple Displacement Amplification (MDA) using bacteriophage phi29 polymerase with random primers allows DNA synthesis in amounts compatible with the downstream use of DNA microarrays [1]. Moreover, MDA has the potential to amplify the whole DNA genome (whole genome amplification, WGA) of target pathogens in the presence of contaminating DNA. WGA technology has become a useful upfront amplification method for many biomedical applications, such as microsatellite analysis, single nucleotide polymorphism (SNP) detection and comparative genomic hybridization (CGH) to microarrays. We recently showed that WGA can now be used for DNA viral pathogen detection from clinical samples using resequencing microarrays [2]. Indeed, resequencing technology using microarrays is very promising for bacterial and viral pathogen detection and identification, as well as for the determination of their pathogenicity profile [2-5]. However, MDA cannot be used to amplify RNA nor small cDNA obtained from RNA genomes after reverse transcription or small native DNA fragments such as those generated by RT from segmented riboviral genomes. Therefore, MDA has not been previously used with riboviruses. A novel modified MDA approach by Qiagen designated QuantiTect Whole Transcriptome has been developed for Transcriptome studies. We changed the process by using a different reverse transcription strategy containing random primers instead of a mixture of random and oligo-dT primers and a different reverse transcriptase. In this WTA process, after reverse transcription of RNA utilizing random primers, all cDNAs are being ligated together into longer linear chains allowing concatenated cDNAs from small RNA fragments to be used as templates for MDA. We applied WTA to viral RNA and demonstrated that WTA is applicable for cDNA amplification of a whole range of RNA virus genomes, prior to DNA hybridization based techniques. About half a dozen approaches have been developed for random whole genome amplification upstream of SNP detection methods (e.g. Omniplex^® ^technology [6], DOP-PCR [7,8], LA-PCR [9,10], PCR with universal linker [11] and T7 based linear amplification for genomic DNA [12]). Among them, WGA and Random Amplification (RA) based on random RT and random PCR are the most widespread techniques for the detection and identification using DNA microarrays. In this study, we chose to compare WTA and RA. The final DNA yields generated by RA and by WTA, in the absence or presence of prokaryotic DNA, were evaluated by quantitative PCR (qPCR). The accuracy of identification by high-density microarrays was also compared between RA and WTA processes.

## Methods

### RNA extraction

Total RNA from brain biopsies (5–10 mg) were obtained using 1 ml of TRI Reagent (Molecular Research Center) according to the manufacturer's instructions. Other RNA extraction was performed using QIAamp Viral RNA Mini Kit (Qiagen) according to the manufacturer's instructions.

### Synthesis of viral RNA complementary strand

The complementary strand (cDNA) of extracted viral RNA was performed in a 200 μl tube. The primer used for RA was described by Wang *et al *[13] whereas cDNA synthesis for WTA was performed with random hexamer primers at the same concentration except for the influenza B virus experiment where the final concentration was diluted 10-fold. A mix with 8 μl of RNA, 1 μl of primer (50 μM) and 1 μl of dNTPs (10 mM) was incubated at 75°C for 5 min, cooled on ice for 5 min. Then, 10 μl of 2 × enzyme mix were added. This enzyme mix was composed of 2 μl of 10 × RT Buffer for SSIII (Invitrogen Inc.), 4 μl of 25 mM MgCl_2_, 2 μl of 0.1 M DTT, 1 μl of 40 U/μl RNaseOUT (Invitrogen Inc.), 1 μl of Reverse Transcriptase SuperScript III (Invitrogen Inc.) and 0.5 μl of DMSO (Sigma-Aldrich). The final mix was submitted to the following steps: 25°C for 10 min, 45°C for 90 min and 95°C for 5 min. All cDNAs were stored at -20°C or immediately used.

### Viral RNA amplification based on the Random Amplification method (RA)

After the synthesis of the complementary strand, a second-strand DNA synthesis was carried out with the addition of 10 μl of Klenow mix, consisting of 3 μl of 10 × Klenow Buffer, 2 μl of dNTP (0.5 mM each) and 1 μl of Klenow DNA polymerase I (Biolabs). The final 30 μl mix was incubated at 20°C for 20 min and at 95°C for 5 min. Subsequently, 15 μl of the resulting double stranded DNA was used as template for a 40 cycle PCR with Primer E as previously described by Wang *et al *[13] except the addition of 0.5 μl of DMSO and the use of TaKaRa DNA polymerase (5 U/μl) instead of *Taq *DNA polymerase.

### Viral RNA amplification based on "Whole Transcriptome Amplification" kit (WTA)

Viral RNA amplification was performed as described in the protocol of the QuantiTect Whole Transcriptome Kit (Qiagen) except for the cDNA synthesis step. It was replaced by the reverse transcription protocol as described above. The two following steps were performed according to the manufacturer's instructions (Qiagen).

### Co-amplification of RNA and DNA pathogens

The amplification of viral RNA and bacterial DNA as mentioned above was based on the WTA amplification process as described above except that, after the ligation step, a nucleic acid denaturation was performed by adding 2 μl of denaturating solution available in Repli-g MIDI kit (Qiagen) and incubated for 3 min at room temperature. After that, the final amplification step was performed according to the manufacturer's instructions (Qiagen).

### Identification and quantification by RT-qPCR for Rift Valley Fever Virus

To identify Rift Valley Fever Virus (RVFV), RT-qPCR was performed with the primers published by Drosten *et al*. [14] but using LC RNA Amplification kit SYBRGreen I (Roche Diagnostic) and different RT and PCR cycling conditions. The detection and quantification involved the following steps: reverse transcription at 55°C for 10 min, initial denaturation at 95°C for 30 s, and 45 cycles with 95°C for 5 s and 72°C for 10 s. Fluorescence was read at the combined annealing-extension step at 72°C.

### Identification and quantification by qPCR for Staphylococcus aureus

The LightCycler instrument (Roche Diagnostics) was used to amplify a 197 bp region of the *S. aureus nucA *gene with LC FastStart DNA Master^PLUS ^SYBRGreen kit. PCR was performed in a total volume of 20 μl containing 4 μl of Master mixture including *Taq *polymerase, reaction buffer, and a deoxynucleoside triphosphate mixture; 10.6 μl of pure water and 0.5 μM each of forward (5' GACTATTATTGGTTGATACACCTG 3') and reverse (5' GCCTTGACGAACTAAAGCTTC 3') primers. After distribution of 15 μl of the master mixture, 5 μl of diluted template DNA solution was added to each glass capillary (Roche Diagnostics), centrifuged, and placed in the LightCycler sample carousel. LightCycler amplification involved a first denaturation at 95°C for 10 min, followed by amplification of the target DNA for 50 cycles (95°C for 10 s, 53°C for 20 s, and 72°C for 10 s) with a temperature transition rate of 20°C/s. The fragment amplification step is followed by a melting curve analysis to an increase from 50°C to 95°C at a rate of 0.1°C/s.

### Quantification of amplified DNA

After purification, the DNA obtained was quantified using "Qubit Quantitation Platform" either with the Quant-iT dsDNA HS Assay/Quant-iT dsDNA BR Assay kits for DNA or the Quant-iT RNA Assay kit for RNA as recommended by the manufacturer (Invitrogen^®^).

### Hybridization to microarrays

DNA amounts obtained after amplification were quantified by Quantit BR and Quantit HS (Invitrogen Inc.), for WTA and RA, respectively. The same quantity of DNA was fragmented (GeneChip^® ^Resequencing Assay Kit, Affymetrix Inc.) and labelled according to the GeneChip^® ^Mapping 100 K Assay Manual (Affymetrix Inc.). Microarray hybridization was conducted at 45°C and array processing was carried out according to the protocol recommended by the manufacturer (Affymetrix Inc.) as previously described [2]. All experiments described in these studies were carried out using independent duplicates.

### Data Analysis

The raw image file (.DAT) obtained after scanning the microarray was analyzed using the Affymetrix GeneChip Operating Software (GCOS) to produce a simplified file format (.CEL) with intensities assigned to each of the corresponding probe positions. Next, the Affymetrix GeneChip Sequence Analysis Software (GSEQ), which contains a derivative of the ABACUS algorithm [15], uses the probe intensities to call the bases along genetic fragments included on the microarray, outputting the result in a FASTA file. The analysis parameters were optimized in order to obtain the best call rate value while minimising the rate of resequencing error. The call rate for a fragment is simply the ratio of called bases to the total number of bases expressed as a percentage. More details concerning data analysis are described in Berthet *et al *[2].

## Results

### DNA yields and amplification factors for WTA and RA

Phi29 polymerase requires DNA templates larger than 2 kb, which exceeds the size of the smallest RNA strands of segmented RNA viruses (e.g. influenza viruses). To address this shortcoming, a target cDNA ligation step is performed prior to MDA amplification resulting in the WTA protocol. In this work, WTA was compared to a RA protocol as described in the Material and Methods (M&M). The DNA yields obtained using WTA and RA methods were compared for three different viral RNA genomes. We tested (i) a viral genome fragmented into small segments (influenza B virus: 14 kb and 8 segments), (ii) a fragmented middle size genome (Rift Valley Fever Virus (RVFV): 12 kb and 3 segments) and (iii) a large viral genome (Severe Acute Respiratory Syndrome CoronaVirus (SARS-CoV): 29 kb and 1 segment), which are representative of the extreme diversity in RNA viral genome size. WTA DNA yields were 1.21 ± 0.06 μg/μl, 0.98 ± 0.27 μg/μl and 1.42 ± 0.08 μg/μl, respectively. These yields greatly exceed those observed with RA (0.02 ± 0.01 μg/μl, 0.06 ± 0.02 μg/μl and 0.012 ± 0.004 μg/μl, respectively). It is noteworthy that, in the absence of a DNA template, water controls amplified by WTA yield as much as 0.9 μg/μl of non-specific DNA. This spurious amplification is probably due to priming artefacts or *E. coli *residual DNA from the production process of one of the kit enzymes. However, in the presence of a DNA template, such as *S. aureus *DNA, the proportion of non-specific DNA dramatically decreases to undetectable levels using previously described DNA microarray techniques [2].

**Figure 1:**
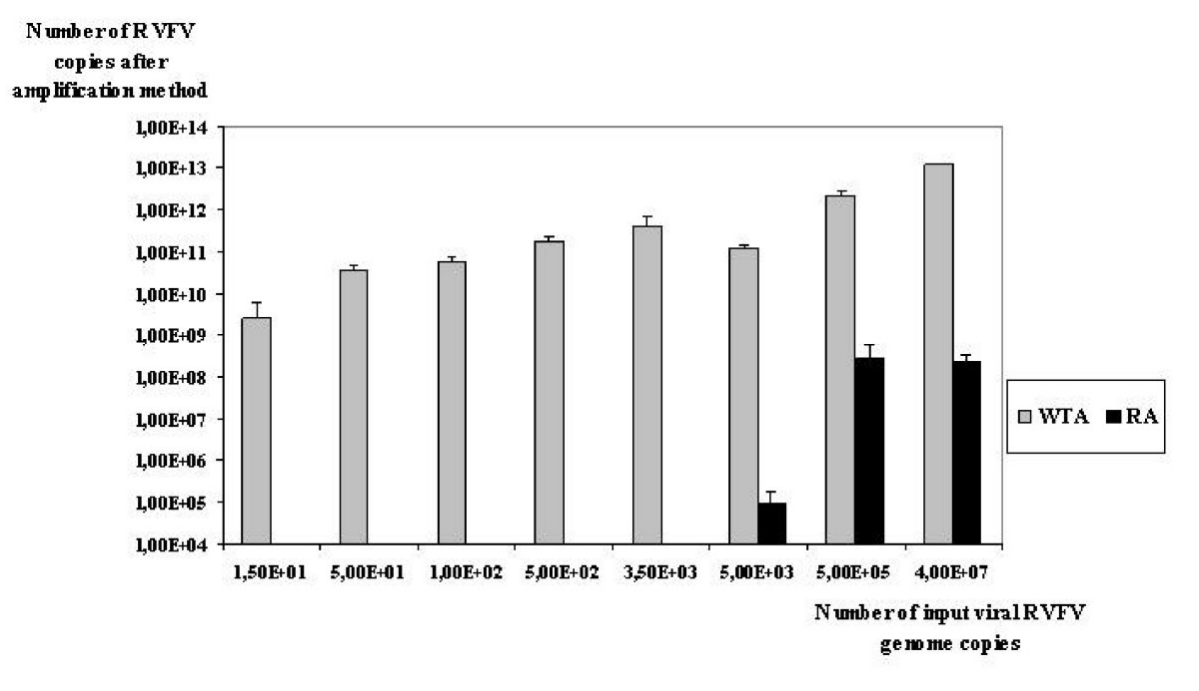
**Comparison of amplification factor between Whole Transcriptome Amplification (WTA) and Random Amplification (RA) protocols**. The number of RVFV copies after amplification is expressed as a function of the amount of input DNA (in number of genome copies). Amounts of RVFV RNA before and after amplification were estimated by qPCR of RVFV as described in M&M.

**Figure 2:**
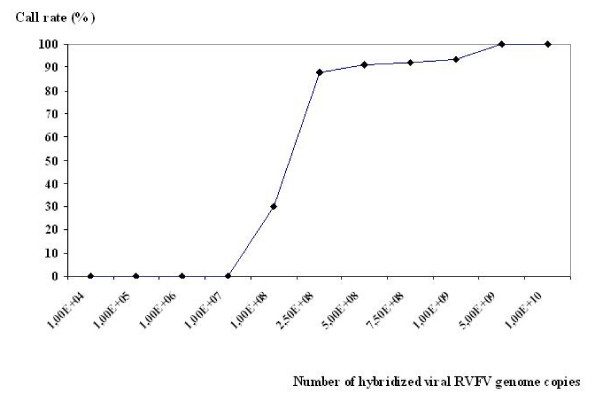
**Mass titration curves with RVFV**. The call rates of the RNA polymerase gene from RVFV are expressed as a function of the amount of input DNA (in number of genome copies).

High amplification factors were obtained starting WTA with various viral genome equivalents. When starting WTA with 15 to 4 × 10^7^copies of RVFV RNA genome copies, the WTA amplification factor ranged from 10^9 ^to 10^6 ^whereas the RA factor varied from 10^3 ^to 10^1 ^respectively (Figure 1). In comparison with RA, the amplification of viral RNA was extremely high irrespective of the amount of RNA genome input. The sensitivity of the detection and identification protocol, based on resequencing microarray technology, was determined by measuring the lowest number of genome copies in the target applied to the microarray. Figure 2 shows the call rate for the RNA polymerase gene of RVFV expressed as a function of the number of genome copies. The call rate is the percentage of bases called by the resequencing algorithm (see M&M). Microarrays required a minimum of ~10^8 ^viral genome copies for gene detection and identification confirmed by BLAST analysis. The high amplification ratio obtained with WTA allowed the detection of RVFV irrespective of the copy number of input viral RNA into the amplification process. These sensitivity levels are compatible with the viral load found in some clinical samples. In sharp contrast, the low amplification ratio observed with RA is irrelevant in clinical situations as it requires a very high viral load for detection by microarrays, *i.e*. to obtain the required ~10^8 ^copies of target sequence for hybridization. To evaluate the potential of WTA in more complex samples where viral RNAs are mixed with cellular RNAs and DNAs, human (n = 2), mouse (n = 10) and dog (n = 2) rabies-infected brain extracts were tested. After hybridization of WTA amplified material on the DNA chip described previously [2], all samples were found positive for the presence of rabies virus with a minimum call rate of 40%.

### Hybridization of viral genomes of various lengths with resequencing microarray

In order to assess the quality of amplified cDNA, hybridization to high density DNA resequencing arrays was performed and the call rate values of the sequences determined through this analysis were compared. To this aim, the viral amplification using WTA was performed with seven viruses representative of the size diversity of viral genomic RNAs, including positive and negative strand genomes as well as non-segmented and segmented genomes (Table 1). These data confirmed that the WTA method amplified viral cDNA from a cell culture supernatant and identified the virus after hybridization. In all cases, virus identification was accomplished using BLAST analysis of DNA sequence determined using the resequencing chip.

**Table 1:** Call rates for seven different viral RNAs using WTA method.

				**Whole Transcriptome Amplification**	
Viral family	Name of virus	Number of fragments (size)	Strand	Call rate (%)	
				
				Average ± SD	Range (Min-Max)

*Coronaviridae*	SARS-CoV^1^	**1 **(29751)	**S +**	99.1 ± 0.3	(98.9–99.3)
*Flaviviridae*	Yellow fever	**1 **(10862)	**S +**	74.4 ± 2.5	(72.6–76.2)
	Dengue type 2	**1 **(10703)	**S +**	59.4 ± 10.7	(51.8–66.9)
*Rhabdoviridae*	VSV^2^	**1 **(11161)	**S -**	96.8 ± 2.1	(95.3–98.3)
	CVS^3^	**1 **(11966)	**S -**	73.6 ± 1.1	(72.8–74.3)
*Bunyaviridae*	Rift valley fever virus	**3 **(12181: 6606*, 3885, 1690)	**S +/-**	100 ± 0	(100–100)
*Orthomyxoviridae*	Influenza virus type B (B/Yamagata/166/98)	**8 **(14289: 2328*, 2352, 2273, 1843,1793, 1504, 1151, 1045)	**S -**	72.3 ± 9.7	(65.4–79.1)

1. Severe Acute Respiratory Syndrome Coronavirus

2. Vesicular Stomatitis Virus

3. Rabies virus: Challenge Virus Strain

S+: Positive RNA genome; S-: Negative RNA genome; S+/-: Negative or ambisense RNA genome

* Genome segment partially tiled on this chip

### Simultaneous amplification of viral RNA and bacterial DNA

In a previous study, we detected and identified DNA from monkeypox virus and *Staphylococcus aureus *in a skin lesion collected from a patient [2]. Similarly, we wanted to check whether a co-infection between a RNA virus and *S. aureus *would lead to such discriminating results, bearing in mind that the ratio between the amount of genetic material of RNA virus and bacteria is even higher. We therefore evaluated the influence of RNA and DNA of diverse origins on amplification results. Simultaneous amplification of viral RNA and bacterial DNA was performed using an optimized WTA protocol as described in the M&M. As shown in Table 2, *S. aureus *DNA amplification was affected by neither the amount of DNA nor the presence of viral cDNA. Similarly, cDNA from viral RNA was amplified, whatever the quantity of viral genome tested. However, the amplification ratio was decreased in the presence of large quantity of *S. aureus *DNA. Considering the sensitivity threshold for the identification of RVFV (Figure 2), this virus would be identified after hybridization on the DNA chip with a high call rate, *i.e*. greater than 80%, in all cases shown in Table 2, except for 1.45 × 10^4 ^copies of viral RNA and 1.29 × 10^6 ^copies of *S. aureus *DNA. Similar results were obtained after the co-amplification of the RVFV cDNA and the DNA of a cowpox virus (data not shown).

**Table 2:** Co-amplification results of RVFV RNA and *S. aureus *DNA.

		*Staphylococcus aureus*	
	
	**Amount input Nucleic Acid**	**1.3 × 10^4 ^copies**	**1.3 × 10^6 ^copies**
	
		Final yield in copies	
		
Rift Valley Fever Virus	**1.5 × 10^4 ^copies**	SA: 2.1 ± 0.9 × 10^9^ RVFV: 2.1 ± 0.3 × 10^10^	SA: 1.4 ± 0.1 × 10^11^ RVFV: 3.8 ± 2.7 × 10^7^
		
	**1.5 × 10^6 ^copies**	SA: 8.2 ± 1.7 × 10^8^ RVFV: 1.37 ± 0.03 × 10^11^	SA: 1.4 ± 0.2 × 10^11^ RVFV: 2.5 ± 0.7 × 10^9^

All the quantifications, both before and after amplification, were determined by qPCR as described in M&M.

SA: *S.aureus*

## Discussion

WTA allowed amplification of a whole range of RNA viral genomes with high sensitivity thereby providing an accurate and highly effective alternative to other random amplification methods. In this study, we changed the WTA method (QuantiTect Whole-Transcriptome Kit, Qiagen) by using a different reverse transcription step and demonstrated unbiased identification of many different viruses using WTA and oligonucleotide resequencing microarray technology. Two viruses belonging to phylogenetically distinct genera (*Vesiculovirus *and *Lyssavirus*) were identified with a call rate of 96.8% and 73.6% respectively. However, amplification output may vary within the same viral genus as illustrated with yellow fever and dengue viruses (*Flavivirus*). The lower call rate obtained for dengue 2 virus might be due to secondary structures in the target sequence, as suggested by the higher call rate obtained with yellow fever virus whose target sequence lacked such structures. These results demonstrate the effect of selected sequences on the final output. As mentioned for the analysis of human, mouse and dog brain samples for the detection of rabies virus, the presence of eukaryotic nucleic acids did not prevent either the amplification using WTA or the downstream identification of the pathogen on DNA microarrays. WTA not only amplified a huge diversity of viral RNAs but also bacterial RNAs such as those transcribed from ribosomal, house-keeping and antibiotic resistance genes, extracted from either a pure bacterial culture or a clinical sample (data not shown). The simultaneous amplification of cDNA from bacterial and viral RNA would be useful for the characterization of many different types of pathogens that cause similar symptoms, such as respiratory syndromes, providing considerable potential for medical use. Samples containing 15 genome copies of viral RNA per target per reaction could be amplified with WTA, to a yield compatible with most of the detection methods used in diagnosis. WTA could thus be used upstream of many pathogen identification protocols for clinical samples (e.g. DNA micro-arrays, liquid DNA arrays, Southern blots, modified qPCR using exonuclease or hybridization probes). DNA polymerization catalyzed by phi29 DNA polymerase is a highly accurate process. Direct sequencing experiments sampling 500 000 bp determined its error rate to be 9.5 × 10^-6 ^[16], making it one of the most accurate polymerases available.

## Conclusion

WTA thus provides an isothermal alternative to random RT-PCR, and could become an invaluable method in diagnostic applications, particularly when used in conjunction with oligonucleotide microarrays.

## Abbreviations

DOP-PCR: Degenerate Oligonucleotide Primer – PCR; MDA: Multiple Displacement Amplification; LA-PCR: linker-adapter-mediated PCR; RA: Random Amplification; RVFV: Rift Valley Fever Virus; SA: *Staphylococcus aureus*; SNP: Single Nucleotide Polymorphism; WGA: Whole Genome Amplification; WTA: Whole Transcriptome Amplification.

## Competing interests

The authors declare that they have no competing interests.

## Authors' contributions

NB, AKR, CB, RS and IL carried out molecular studies. NB, IL and JCM participated in experimental design of the study. NB, IL and JCM participated in drafting the manuscript. NB, AKR, CB, PD, IGO, STC, GK, HB, LD and JCM participated in data analysis. SS and CK participated in the development of WTA kit. All authors added corrections and suggestions to the manuscript. JCM conceived and coordinated the study. All authors read and approved the final manuscript.
